# Evaluation of Cyclic Fatigue of Hyflex EDM, Twisted Files, and ProTaper Gold Manufactured with Different Processes: An In Vitro Study

**DOI:** 10.1155/2021/7402658

**Published:** 2021-07-29

**Authors:** Pooja D. Khandagale, Prashant P. Shetty, Saleem D. Makandar, Pradeep A. Bapna, Mohmed Isaqali Karobari, Anand Marya, Pietro Messina, Giuseppe Alessandro Scardina

**Affiliations:** ^1^Department of Conservative Dentistry and Endodontics, Pacific Dental College and Hospital, Udaipur, Rajasthan, India; ^2^Department of Conservative Dentistry and Endodontics, Pacific Dental College, Debari, Udaipur 313024, India; ^3^Conservative Dentistry Unit, School of Dental Sciences, Universiti Sains Malaysia, Health Campus, 16150 Kubang Kerian, Kota Bharu, Kelantan, Malaysia; ^4^Department of Conservative Dentistry & Endodontics, Saveetha Dental College and Hospitals, Saveetha Institute of Medical and Technical Sciences University, Chennai 600077, Tamil Nadu, India; ^5^Department of Orthodontics, Faculty of Dentistry, University of Puthisastra, Phnom Penh, Cambodia; ^6^Department of Surgical Oncological and Stomatological Disciplines, University of Palermo, Palermo, Italy

## Abstract

**Introduction:**

The main aims of root canal instrumentation are to provide an environment that will lead to healing and to provide a root canal shape that is comfortable to clean and seal. When working with rotary endodontic instruments, the most significant concerns are that the instrument might fracture in the root canal, thus affecting the treatment outcome. Hence, it is of immense importance to know which file systems have more cyclic fatigue resistance. *Methodology*. This study evaluated the effect of the curved segment length of the artificial canal (the arch), and the number of cycles necessary in fracture of Hyflex EDM, Twisted files, and ProTaper Gold were recorded. Sixty NiTi rotary instruments of 25 mm length (Hyflex EDM (20), Twisted files (20), and ProTaper Gold (20)) were tested in a metal block with simulated canal having 90° angle of curvature. The study was performed with a specific radius and degree of curvature, i.e., 8 mm radius and 90⁰ angle of curvature, and data obtained were subsequently subjected to statistical evaluation using one-way analysis of variance and Tukey's post hoc test.

**Result:**

The Hyflex EDM (774.29) exhibited the maximum cyclic fatigue resistance compared to Twisted files (654.875) and ProTaper Gold (375.575). A statistically significant difference was observed between the tested groups.

**Conclusion:**

The Hyflex EDM files showed the highest cyclic fatigue resistance, followed by Twisted files and ProTaper Gold files.

## 1. Introduction

The main aim of root canal therapy is to prevent and treat the diseases of the dental pulp and periapical tissues [1]_._ The bacterial incursion of root canals primarily causes apical periodontitis. Hence, the treatment focuses on removing microbes from the root canal system and preventing reinfection [2]. In an extensive study of the quality of endodontic treatment in a Belgian population, De Moor et al. evaluated the periapical conditions in 4617 teeth of 206 adults using panoramic radiographs. Of all the teeth, 6.8% were endodontically treated. Comparison of periapical status showed that apical periodontitis was found in 6.6% of all teeth and 40.4% of all root-filled teeth. An inadequate level of the root canal filling was registered in over 50% of these teeth [3].

The use of irrigating solutions with prominent antimicrobial action is an essential aid in mechanical preparation in requisition to reduce microbial load further. To achieve these goals, mechanical shaping of root canal systems has been accomplished with a wide range of methods and instruments along with various irrigating solutions [4]. Canal instrumentation by hand was the first technique to be applied and is still being used now. A study conducted by Iqbal et al. revealed that the prevalence of instrument separation in hand instrumentation (0.17%) is less as compared with rotary instrumentation (0.67%) [5].

The introduction of nickel-titanium (NiTi) rotary instruments in 1988 by Walia et al. [6] significantly changed endodontics, offering an efficient and systematic instrumentation method. Rotary NiTi systems available today are quite diverse, all in their design, cross section, configuration, and uses.

Despite improvements in the manufacturing process, instrument fracture is still a primary concern when using NiTi rotary instruments. The 2 main reasons for fracture of rotary NiTi files are torsional stress and cyclic fatigue [7]. Cyclic fatigue takes place when the file is freely rotating in a canal and flexes until a fracture occurs. This happens because of alternating compression/tension cycles that files are subjected to when tensed in the area of the greatest curvature of the canal [7]. Various strategies reported that would enhance instrument resistance to cyclic fatigue comprise development in the manufacturing method or new alloys that would offer improved mechanical properties.

Recently advanced manufacturing processes for NiTi endodontic instruments have been developed to overwhelm these drawbacks. The novel NiTi endodontic instruments have been treated with different surface treatments and advanced metallurgy to improve their mechanical properties [8]. In the present study, three different NiTi rotary files designed by different manufacturing processes were used. The replicated canals operated in this research systematized the working nature of each instrument. Although the simulated canals did not replicate the in vivo situations, it allowed a comparative analysis of different instruments in a systematized nature.

Hence, the purpose of the current study was to evaluate the cyclic rotations and time required in fracture of Hyflex EDM, Twisted files, and ProTaper Gold in simulated 90° curved canals.

## 2. Methodology

The sample size calculation was performed using a G-power 3.1.9.4. The sample size obtained was 57, with each group containing 19 samples.

Sixty NiTi rotary instruments of 25 mm length (Hyflex EDM (20), Twisted files (20), and ProTaper Gold (20)) were tested in a metal block with a simulated canal having 90° angle of curvature. The NiTi files used in this study were as follows:

  Group I: Hyflex EDM files (one file system, size 25) developed from a technology called “Electrical Discharge Machining”
  Group II: Twisted files (size 25) treated with R-phase heat treatment technology
  Group III: ProTaper Gold (F2) manufactured from enhanced heat treatment technology

The instruments were divided into three groups based on the manufacturing process. The apical size of the instrument at D used in the study was standardized in all the groups. The study was performed with a specific radius and degree of curvature, i.e., 8 mm radius and 90° angle of curvature. All the files were rotated at a particular speed and torque as recommended by the manufacturer in a custom-made experimental setup that simulated curved canals. The applied torque was 3 N cm for all the file systems. The recommended speed for each file system as per the manufacturer instructions was as follows: Hyflex EDM-500 RPM, Twisted files-500 RPM, and ProTaper Gold-300 RPM. The 36.8 mm × 25.4 mm × 9.5 mm block was made from a 300-series stainless-steel block and simulated the artificial canal. An artificial canal within a stainless-steel block was milled using a diode laser with the aid of computer program, which reproduced the instrument size and preserved the instrument trajectory adapted to the parameters selected. The size of the artificial canal was increased by 0.2 mm to the original size of the instrument.

**Figure 1:**
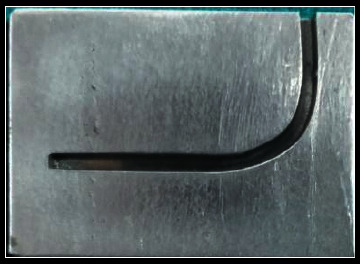
Custom-made metal block simulating curved canal with a degree of curvature 90°.

**Figure 2:**
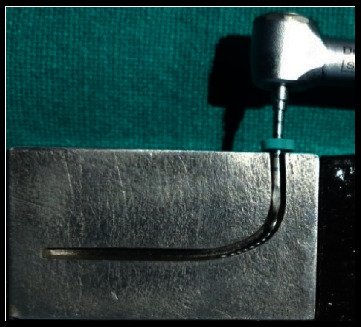
Rotary files instrumented in the simulated curved canals until fracture.

The metal testing block consisted of a 1.5 mm-wide simulated canal with 90⁰ curvature (Figure 1). The instruments were rotated in an electric torque-controlled motor with a 20 : 1 gear handpiece (Figure 2). Before rotation, the instruments were introduced into the canals to the full working length by moving the block towards the handpiece. The diameter of the canals was larger than the instruments, allowing their free rotation. To reduce the friction while contacting the metal canal walls, glycerin was used as lubricant.

The files used in the study were instrumented up to 20 mm working length for all the groups. Each instrument was allowed to rotate until a fracture occurred. The instrument fracture was visually detected, the time to fracture was recorded using a digital stopwatch, and timing was stopped as fracture was detected visually/or audibly.

The number of cycles to fracture (NCF) was calculated according to the following formula:

(1) $$\begin{array}{c} \text{NCF}=\frac{\text{time}\left(\sec\right)\times \text{speed}}{60}. \end{array}$$

Mean values and standard deviations of NCF were calculated for each system. All data were subsequently subjected to statistical evaluation using one-way analysis of variance and Tukey's post hoc test.

Each system of NCF was calculated for mean values and standard deviations. The data obtained were subjected to statistical evaluation using one-way ANOVA and Tukey's post hoc test. One-way analysis of variance (ANOVA) is done to test several means (more than two) at a time. Tukey's post hoc test is used for two by two comparisons of the groups (Tables 1 and 2).

## 3. Results

The mean no. of cycles required for groups I, II, and III were 774.29, 654.875, and 375.575, respectively. The difference in the no. of cycles required for fracture among all the groups was statistically significant. Pairwise comparison of the number of cycles required for fracture showed statistically significant differences among all the three groups.

Pairwise comparison of no. of cycles required for fracture showed statistically significant difference (*p*=0.001) between Hyflex EDM and Twisted files groups. Pairwise comparison of no. of cycles required for fracture showed significant difference (*p*=0.001) between Hyflex EDM and ProTaper Gold files. Pairwise comparison of no. of cycles required for fracture showed significant difference (*p*=0.001) between Twisted files group and ProTaper Gold files group.

## 4. Discussion

The introduction of rotary NiTi files in the field of endodontics has significantly changed the outcome of endodontic therapy. Rotary file systems have greatly enhanced the level of precision and speed of the endodontic treatment. Apart from advantages, the rotary files also possess few disadvantages. The file separation may take place inadvertently, which compromises the outcome of the endodontic rehabilitation and healing of the periradicular tissues [9].

Several factors contribute to the fracture of the rotary instrument. These include handling by the operator, usage, anatomy of the root canal, and design of NiTi rotary instruments. Because of these, various studies have been performed to investigate the reasons and physics of the instrument fracture [10]. The mechanism of rotary instrument separation was reported as a torsional failure and cyclic fatigue fracture [11]. Cyclic flexural fatigue occurs when an instrument rotates in a curved canal by repetitive compressive and tensile stresses, and torsional failure occurs when the instruments' tip is locked or jammed in the canal, but the shank of the file keeps rotating [12].

It has been reported that there was a high incidence of torsional failure (56%) than cyclic fatigue (44%) after prolonged use of rotary NiTi files [7, 10]. It is difficult to avoid the fact that both the flexural and torsional stresses occur concurrently, but until now, only some reports have associated them with each other. It has been reported that cyclic flexural straining substantially decreased the torsional resistance, predominantly of the preloaded instruments at three-fourths of their lifetime [13].

In the current study, rotary files designed with three different manufacturing processes were used to estimate their cyclic fatigue resistance. Hyflex EDM (Coltene/Whaledent, Switzerland) instruments are manufactured by means of electrical discharge machining (EDM) process, wherein workpieces are machined by generating a potential between the workpiece and the tool. The novelty of the HyFlex EDM owes its unique properties to a breakthrough technology called “Electrical Discharge Machining.” This innovative manufacturing process uses spark erosion to harden the surface of the NiTi file, resulting in superior fracture resistance and improved cutting efficiency. Just like HyFlex CM files, HyFlex EDM files offer trusted controlled memory effect and regenerative properties [14].

The Twisted files (Kerr Dental, United States) are created by converting a raw NiTi wire from the austenite phase into the R-phase by means of a thermal procedure [15]. The ProTaper Gold files (Dentsply, Tulsa Dental Specialties, OK, USA) were created with proprietary superior metallurgy through heat treatment technology [16]. Most of the previous studies were performed by using metal tubes that were cylindrical, of at least 1 mm diameter with different angles of curvature and radius [17], whereas others have used an inclined metal block to mock up different angles [18].

Therefore, in the present study, an artificial standardized canal was created in a 300-series stainless-steel metal block to analyse the cyclic fatigue resistance. Uslu et al. [19] looked at different radii of curves and instrument sizes and found that time to fracture decreased as the radius of curvature decreased. Researchers also investigated the effect of the angle and radius of canal curvature on cyclic flexural fatigue, and it was discovered that an increasing angle and decreasing radius of canal curvature significantly reduced the number of rotations the instrument could withstand before fracture. Therefore, in the present study, 8 mm radius and 90° angle of curvature of the metal block were tested.

Various studies have found that instruments rotated at greater speed have more possibility of getting fractured when compared to the instruments that were used at lower rotational speeds, which are less likely to get fractured [20, 21].

In few studies, it was observed that the rotational speed of the instruments did not seem to influence the frequency of file fracture, but some studies say that it may cause varying test conditions, different operators, and different file systems [22]. In the present study, instruments were operated as per manufacturers' instructions. Torque might affect the frequency with which instrument breaks. When an Endomotor with high torque is used, it is likely to surpass the instrument's fracture point within the canal, so a possible solution would be to utilize an endodontic motor with low torque, which would run within the maximum permissible torque limit for each rotary instrument, thus avoiding the fracture [23]. The present study used a torque value of 3 N cm, which was standardized for all three groups. In various studies, lubricants in the form of petroleum jelly [24], cold spray [7], RC-Prep [25], and glycerin [16] were used in the stimulated canals to prevent overheating of endodontic files during rotations. In the present study, glycerin was used as a lubricant.

**Figure 3:**
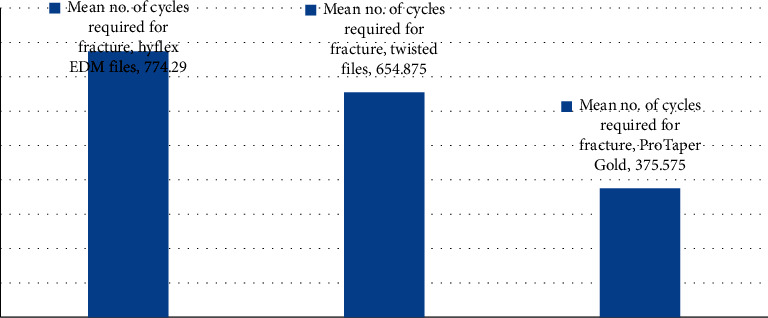
Mean number of cycles required for fracture.

The result of the present study revealed that there was an increase in cyclic fatigue resistance in samples instrumented by Hyflex EDM (Group A), when compared to Twisted files (Group B) and ProTaper Gold (Group C) (Figure 3). The mean value recorded in group A, group B, and group C were 774.9 N, 654.875 N, and 375.575 N, respectively. The Hyflex EDM files showed the highest cyclic fatigue resistance, followed by Twisted files and ProTaper Gold files. A statistically significant difference was witnessed among all the test groups evaluated (*p*=0.001^*∗*^).

The maximum fatigue resistance with Hyflex EDM (Group A) could be attributed to its EDM process, unique cross-section design, and greater austenite finish temperature (more than 370°C), which has both mixtures of austenite and martensitic structure at room temperature [26]. Martensitic structure is less rigid compared to austenite and files produced by means of CM wires have more martensite at the cost of austenite, and consequently, this affects their fatigue properties [27] exhibits the flexibility and fracture resistance up to 700%. Elizabeth et al. [28] undertook a study where Hyflex EDM exhibited maximum cyclic fatigue resistance in comparison with Twisted files and Wave One Gold files. The present study's findings must be proven by additional investigation, which assesses other scientifically significant mechanical properties of the instruments. Further studies are also required to compare in vitro and in vivo fatigue resistances of NiTi rotary instruments.

## 5. Conclusion

The conclusions were drawn within the limitations of the study. Hyflex EDM showed the highest cyclic fatigue resistance amongst all the tested groups. The least cyclic fatigue resistance was found in ProTaper Gold files.

## Figures and Tables

**Table 1:** Comparison of no. of cycles required for fracture among study groups using the one-way ANOVA test.

Groups	*N*	Mean	Std. deviation	95% confidence interval for mean		*p* value
				Lower bound	Upper bound	
Hyflex EDM files	20	774.29	64.17	744.26	804.32	0.001^*∗*^
Twisted files	20	654.875	85.33	614.94	694.81	
ProTaper Gold	20	375.575	19.84	366.29	384.86	

**Table 2:** Pairwise comparison of no. of cycles required for fracture using Tukey's post hoc test.

Groups	Comparison group	Difference	*p* value
Hyflex EDM files	Twisted files	119.415	0.001^*∗*^
Hyflex EDM files	ProTaper Gold files	398.715	0.001^*∗*^
Twisted files	ProTaper Gold files	279.300	0.001^*∗*^

## Data Availability

The data used to support the findings of this study are available from the corresponding author upon request.
